# Power priors for replication studies

**DOI:** 10.1007/s11749-023-00888-5

**Published:** 2023-09-21

**Authors:** Samuel Pawel, Frederik Aust, Leonhard Held, Eric-Jan Wagenmakers

**Affiliations:** 1 Epidemiology, Biostatistics and Prevention Institute (EBPI), Center for Reproducible Science (CRS), University of Zurich, Zurich, Switzerland; 2 Department of Psychological Methods, University of Amsterdam, Amsterdam, The Netherlands

**Keywords:** Bayes factor, Bayesian hypothesis testing, Bayesian parameter estimation, Hierarchical models, Historical data, 62Fxx Parametric inference, 62Kxx Design of statistical experiments, 62Jxx Linear inference, regression, 62Lxx Sequential statistical methods, 62Pxx Applications of statistics

## Abstract

The ongoing replication crisis in science has increased interest in the methodology of replication studies. We propose a novel Bayesian analysis approach using power priors: The likelihood of the original study’s data is raised to the power of $\alpha$, and then used as the prior distribution in the analysis of the replication data. Posterior distribution and Bayes factor hypothesis tests related to the power parameter $\alpha$ quantify the degree of compatibility between the original and replication study. Inferences for other parameters, such as effect sizes, dynamically borrow information from the original study. The degree of borrowing depends on the conflict between the two studies. The practical value of the approach is illustrated on data from three replication studies, and the connection to hierarchical modeling approaches explored. We generalize the known connection between normal power priors and normal hierarchical models for fixed parameters and show that normal power prior inferences with a beta prior on the power parameter $\alpha$ align with normal hierarchical model inferences using a generalized beta prior on the relative heterogeneity variance $I^2$. The connection illustrates that power prior modeling is unnatural from the perspective of hierarchical modeling since it corresponds to specifying priors on a relative rather than an absolute heterogeneity scale.

## Introduction

Power priors form a class of informative prior distributions that allow data analysts to incorporate historical data into a Bayesian analysis (Ibrahim et al. 2015). The most basic version of the power prior is obtained by updating an initial prior distribution with the likelihood of the historical data raised to the power of $\alpha$, where $\alpha$ is usually restricted to the range from zero (i.e., complete discounting) to one (i.e., complete pooling). As such, the power parameter $\alpha$ specifies the degree to which historical data are discounted, thereby providing a quantitative compromise between the extreme positions of completely ignoring and fully trusting the historical data.

One domain where historical data are per definition available is the analysis of replication studies. One pertinent question in this domain is the extent to which a replication study has successfully replicated the result of an original study (National Academies of Sciences, Engineering, and Medicine 2019). Many methods have been proposed to address this question (Bayarri and Mayoral 2002a; Verhagen and Wagenmakers 2014; Johnson et al. 2016; Etz and Vandekerckhove 2016; van Aert and van Assen 2017; Ly et al. 2018; Hedges and Schauer 2019; Mathur and VanderWeele 2020; Held 2020; Pawel and Held 2020, 2022; Held et al. 2022, among others). Here we propose a new and conceptually straightforward approach, namely to construct a power prior for the data from the original study, and to use that prior to draw inferences from the data of the replication study. The power prior approach can accommodate two common notions of replication success: First, the notion that the replication study should provide evidence for a genuine effect. This can be quantified by estimating and testing an effect size $\theta$, typically by assessing whether there is evidence that $\theta$ is different from zero. Second, the notion that the data from the original and replication studies should be compatible. This can be quantified by estimating and testing of the power parameter $\alpha$. Values close to $\alpha = 1$ indicate compatibility as there is a complete pooling of both data sets, and values close to $\alpha = 0$ indicate incompatibility as the original data are completely discounted.

Below we first show how power priors can be constructed from data of an original study under a meta-analytic framework (Sect. 2). We then shown how the power prior can be used for parameter estimation (Sect. 2.1) and Bayes factor hypothesis testing (Sect. 2.2). Throughout, the methodology is illustrated by application to data from three replication studies which were part of a large-scale replication project (Protzko et al. 2020). In Sect. 3, we explore the connection to the alternative hierarchical modeling approach for incorporating the original data (Bayarri and Mayoral 2002a, b; Pawel and Held 2020), which has been previously used for evidence synthesis and compatibility assessment in replication settings. In doing so, we identify explicit conditions under which posterior distributions and tests can be reverse-engineered from one framework to the other. Essentially, power prior inferences using the commonly assigned beta prior on the power parameter $\alpha$ align with normal hierarchical model inferences if either a generalized F prior is assigned to the between-study heterogeneity variance $\tau ^2$ which scales with the variance of the original data, or if a generalized beta prior is assigned to the relative heterogeneity $I^2$. This perspective also explains the observed difficulty of making conclusive inferences about the power parameter $\alpha$, as it is difficult to make inferences about a variance from two observations alone, and also because the commonly assigned beta prior on $\alpha$ is entangled with the variance from the data.

## Power prior modeling of replication studies

Let $\theta$ denote an unknown effect size and $\hat{\theta }_{i}$ an estimate thereof obtained from study $i \in \{o, r\}$ where the subscript indicates “original” and “replication”, respectively. Assume that the likelihood of the effect estimates can be approximated by a normal distribution

$$\begin{aligned} \hat{\theta }_{i} \,\vert \,\theta \sim {{\,\textrm{N}\,}}(\theta , \sigma ^{2}_{i}) \end{aligned}$$

with $\sigma _{i}$ the (assumed to be known) standard error of the effect estimate $\hat{\theta }_{i}$. The effect size may be adjusted for confounding variables, and depending on the outcome variable, a transformation may be required for the normal approximation to be accurate (e. g.,  a log-transformation for an odds ratio effect size). This is the same framework that is typically used in meta-analysis, and it is applicable to many types of data and effect sizes (Spiegelhalter et al. 2004, chapter 2.4). There are, of course, situations where the approximation is inadequate and modified distributional assumptions are required (e. g.,  for data from studies with small sample sizes and/or extreme effect sizes).

The goal is now to construct a power prior for $\theta$ based on the data from the original study. Updating of an (improper) flat initial prior $f(\theta ) \propto 1$ by the likelihood of the original data raised to a (fixed) power parameter $\alpha$ leads to the normalized power prior

1 $$\begin{aligned} \theta \,\vert \,\hat{\theta }_{o}, \alpha \sim {{\,\textrm{N}\,}}\left( \hat{\theta }_{o}, \sigma ^{2}_{o}/\alpha \right) \end{aligned}$$

as first proposed by Duan et al. (2005), see also Neuenschwander et al. (2009). There are different ways to specify $\alpha$. The simplest approach fixes $\alpha$ to an *a priori* reasonable value, possibly informed by background knowledge about the similarity of the two studies. Another option is to use the empirical Bayes estimate (Gravestock and Held 2017), that is, the value of $\alpha$ that maximizes the likelihood of the replication data marginalized over the power prior. Finally, it is also possible to specify a prior distribution for $\alpha$, the most common choice being a beta distribution $\alpha \,\vert \,x, y \sim {{\,\textrm{Be}\,}}(x, y)$ for a normalized power prior conditional on $\alpha$ as in (1). This approach leads to a joint prior for the effect size $\theta$ and power parameter $\alpha$ with density

2 $$\begin{aligned} f(\theta , \alpha \,\vert \,\hat{\theta }_o, x, y) = {{\,\textrm{N}\,}}(\theta \,\vert \,\hat{\theta }_o, \sigma ^2_o/\alpha ) \, {{\,\textrm{Be}\,}}(\alpha \,\vert \,x, y) \end{aligned}$$

where ${{\,\textrm{N}\,}}(\cdot \,\vert \,m, v)$ is the normal density function with mean *m* and variance *v*, and ${{\,\textrm{Be}\,}}( \cdot \,\vert \,x, y)$ is the beta density with parameters *x* and *y*. The uniform distribution ($x = 1$, $y = 1$) is often recommended as the default choice (Ibrahim et al. 2015). We note that $\alpha$ does not have to be restricted to the unit interval but could also be treated as a relative precision parameter (Held and Sauter 2017). We will, however, not consider such an approach since power parameters $\alpha > 1$ lead to priors with more information than what was actually supplied by the original study.

### Parameter estimation

Updating the prior (2) with the likelihood of the replication data leads to the posterior distribution

3 $$\begin{aligned} f(\alpha , \theta \,\vert \,\hat{\theta }_{r}, \hat{\theta }_{o}, x, y) =&\frac{{{\,\textrm{N}\,}}(\hat{\theta }_{r}\,\vert \,\theta , \sigma ^{2}_{r}) \, {{\,\textrm{N}\,}}(\theta \,\vert \,\hat{\theta }_{o}, \sigma ^{2}_{o}/\alpha ) \, {{\,\textrm{Be}\,}}(\alpha \,\vert \,x, y)}{ f(\hat{\theta }_{r} \,\vert \,\hat{\theta }_{o}, x, y)}. \end{aligned}$$

The normalizing constant

4 $$\begin{aligned} f(\hat{\theta }_{r} \,\vert \,\hat{\theta }_{o}, x, y)&= \int _{0}^{1} {{\,\textrm{N}\,}}(\hat{\theta }_{r}\,\vert \,\hat{\theta }_{o}, \sigma ^{2}_{r} + \sigma ^{2}_{o}/\alpha ) \, {{\,\textrm{Be}\,}}(\alpha \,\vert \,x, y) \, \text {d}\alpha \end{aligned}$$

is generally not available in closed form but requires numerical integration with respect to $\alpha$. If inference concerns only one parameter, a marginal posterior distribution for either $\alpha$ or $\theta$ can be obtained by integrating out the corresponding nuisance parameter from (3). In the case of the power parameter $\alpha$, this leads to

5 $$\begin{aligned} f(\alpha \,\vert \,\hat{\theta }_{r}, \hat{\theta }_{o}, x, y) =&\frac{{{\,\textrm{N}\,}}(\hat{\theta }_{r}\,\vert \,\hat{\theta }_{o}, \sigma ^{2}_{r} + \sigma ^{2}_{o}/\alpha ) \, {{\,\textrm{Be}\,}}(\alpha \,\vert \,x, y)}{f(\hat{\theta }_{r} \,\vert \,\hat{\theta }_{o}, x, y)} \end{aligned}$$

whereas for the effect size $\theta$, this gives

$$\begin{aligned} f(\theta \,\vert \,\hat{\theta }_{r}, \hat{\theta }_{o}, x, y)&= \frac{{{\,\textrm{N}\,}}(\hat{\theta }_{r}\,\vert \,\theta , \sigma ^{2}_{r}) \, \text{ B }(x + 1/2, y)}{ f(\hat{\theta }_{r} \,\vert \,\hat{\theta }_{o}, x, y) \, \sqrt{2\pi \sigma _o^2} \, \text{ B }(x, y)} \,\\&\quad \times M\bigg \{x + 1/2, x + y + 1/2, -\frac{(\hat{\theta }_o - \theta )^2}{2\sigma ^2_o}\bigg \} \end{aligned}$$

with $\text{ B }(z, w) = \int _0^1 t^{z-1}(1 - t)^{w-1} \,\text {d}t = \{\Gamma (z)\Gamma (w)\}/\Gamma (z + w)$ the beta function and $M(a, b, z) = \{\int _0^1 \exp (zt) t^{a-1}(1-t)^{b-a-1} \,\text {d}t\}/\text{B }(b - a, a)$ the confluent hypergeometric function (Abramowitz and Stegun 1965, chapters 6 and 13).

#### Example “Labels”

We now illustrate the methodology on data from the large-scale replication project by Protzko et al. (2020). The project featured an experiment called “Labels” for which the original study reported the following conclusion: *“When a researcher uses a label to describe people who hold a certain opinion, he or she is interpreted as disagreeing with those attributes when a negative label is used and agreeing with those attributes when a positive label is used”* (Protzko et al. 2020, p. 17). This conclusion was based on a standardized mean difference effect estimate $\hat{\theta }_{o} = 0.21$ and standard error $\sigma _{o} = 0.05$ obtained from 1577 participants. Subsequently, four replication studies were conducted, three of them by a different laboratory than the original one, and all employing large sample sizes. Since the same original study was replicated by three independent laboratories, this is an instance of a “multisite” replication design (Mathur and VanderWeele 2020). While in principle it would be possible to analyze all of these studies jointly, we will show separate analyses for each pair of original and replication study as it reflects the typical situation of only one replication study being conducted per original study. Section 4 discusses possible extensions of the power prior approach for joint analyses in multisite designs.

**Fig. 1:**
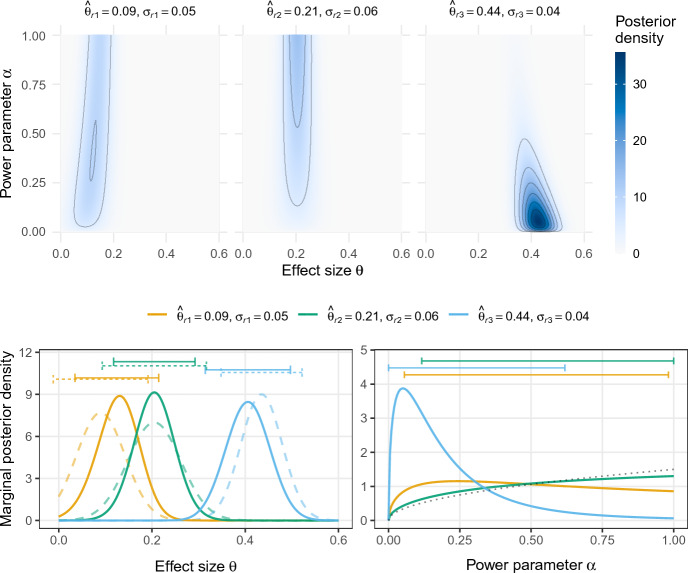
Joint (top) and marginal (bottom) posterior distributions of effect size $\theta$ and power parameter $\alpha$ based on data from the “Labels” experiment (Protzko et al. 2020). The dashed lines depict the posterior density for the effect size $\theta$ when the replication data are analyzed in isolation without incorporation of the original data. The horizontal error bars represent the corresponding 95% highest posterior density credible intervals. The dotted line represents the limiting posterior density of the power parameter $\alpha$ for perfectly agreeing original and replication studies

Figure 1 shows joint and marginal posterior distributions for effect size $\theta$ and power parameter $\alpha$ based on the results of the three external replication studies and a power prior for the effect size $\theta$ constructed from the original effect estimate $\hat{\theta }_{o} = 0.21$ (with standard error $\sigma _{o} = 0.05$) and an initial flat prior $f(\theta ) \propto 1$. The power parameter $\alpha$ is assigned a uniform ${{\,\textrm{Be}\,}}(x = 1, y = 1)$ prior distribution. The first replication found an effect estimate which was smaller than the original one ($\hat{\theta }_{r1} = 0.09$ with $\sigma _{r1} = 0.05$), whereas the other two replications found effect estimates that were either identical ($\hat{\theta }_{r2} = 0.21$ with $\sigma _{r2} = 0.04$) or larger ($\hat{\theta }_{r3} = 0.44$ with $\sigma _{r3} = 0.06$) than that reported in the original study. This is reflected in the marginal posterior distributions of the power parameter $\alpha$, shown in the bottom right panel of Fig. 1. That is, the marginal distribution of the first replication (yellow) is slightly peaked around $\alpha = 0.2$ suggesting some incompatibility with the original study. In contrast, the second replication shows a marginal distribution (green) which is monotonically increasing so that the value $\alpha = 1$ receives the highest support, thereby indicating compatibility of the two studies. Finally, the marginal distribution of the third replication (blue) is sharply peaked around $\alpha = 0.05$ with 95% credible interval from 0 to 0.62 indicating strong conflict between this replication and the original study. The sharply peaked posterior is in stark contrast to the relatively diffuse posteriors of the first and second replications which hardly changed from the uniform prior. This is consistent with the asymptotic behavior of normalized power priors identified in Pawel et al. (2023a); In case of data incompatibility, normalized power priors with beta prior assigned to $\alpha$ permit arbitrarily peaked posteriors for small values of $\alpha$. In contrast, for perfectly agreeing original and replication studies ($\hat{\theta }_{o} = \hat{\theta }_{r}$) there is a limiting posterior for $\alpha$ that gives only slightly more probability to values near one. The limiting posterior is in this case a ${{\,\textrm{Be}\,}}(3/2, 1)$ distribution, whose density is indicated by the dotted line. One can see, that the (green) posterior from the second replication is relatively close to the limiting posterior, despite its finite sample size. Similarly, the corresponding (green) 95% credible interval from 0.12 to 1 suggests that a wide range of very low to very high $\alpha$ values remain credible despite the excellent agreement of original and replication study.

The bottom left panel of Fig. 1 shows the marginal posterior distribution of the effect size $\theta$. Shown is also the posterior distribution of $\theta$ when the replication data are analyzed in isolation (dashed line), to see the information gain from incorporating the original data via a power prior. The degree of compatibility with the replication study influences how much information is borrowed from the original study. For instance, the (green) marginal posterior density based on the most compatible replication ($\hat{\theta }_{r2} = 0.21$) is the most concentrated among the three replications, despite the standard error being the largest ($\sigma _{r2} = 0.06$). Consequently, the 95% credible interval of $\theta$ is substantially narrower compared to the credible interval from the analysis of the replication data in isolation (dashed green). In contrast, the (blue) marginal posterior of the most conflicting estimate ($\hat{\theta }_{r3} = 0.44$) borrows less information and consequently yields the least peaked posterior, despite the standard error being the smallest ($\sigma _{r3} = 0.04$). In this case, the conflict with the original study even inflates the variance of posterior compared to the isolated replication posterior given by dashed blue line. This is, for example, apparent through its 95% credible interval (0.31 to 0.5) being even wider than the credible interval (0.35 to 0.52) based on the analysis of the replication data in isolation.

### Hypothesis testing

In addition to estimating $\theta$ and $\alpha$, we may also be interested in testing hypotheses about these parameters. Let $\mathcal {H}_{0}$ and $\mathcal {H}_{1}$ denote two competing hypotheses, each of them with an associated prior $f(\theta , \alpha \,\vert \,\mathcal {H}_{i})$ and a resulting marginal likelihood obtained from integrating the likelihood of the replication data with respect to the prior

6 $$\begin{aligned} f(\hat{\theta }_r \,\vert \,\mathcal {H}_{i}) = \int {{\,\textrm{N}\,}}(\hat{\theta }_r \,\vert \,\theta , \sigma ^2_r) \, f(\theta , \alpha \,\vert \,\mathcal {H}_{i}) \,\text {d}\theta \,\text {d}\alpha \end{aligned}$$

for $i \in \{0, 1\}$. A principled Bayesian hypothesis testing approach is to compute the Bayes factor

$$\begin{aligned} {{\,\textrm{BF}\,}}_{01}(\hat{\theta }_r) = \frac{\Pr (\mathcal {H}_{0} \,\vert \,\hat{\theta }_r)}{\Pr (\mathcal {H}_{1} \,\vert \,\hat{\theta }_r)} \, \bigg / \, \frac{\Pr (\mathcal {H}_{0})}{\Pr (\mathcal {H}_{1})} = \frac{f(\hat{\theta }_r \,\vert \,\mathcal {H}_{0})}{f(\hat{\theta }_r \,\vert \,\mathcal {H}_{1})} \end{aligned}$$

since it corresponds to the updating factor of the prior odds to the posterior odds of the hypotheses based on the data $\hat{\theta }_{r}$ (first equality), or because it represents the relative accuracy with which the hypotheses predict the data $\hat{\theta }_{r}$ (second equality) (Jeffreys 1939; Good 1958; Kass and Raftery 1995). A Bayes factor ${{\,\textrm{BF}\,}}_{01}(\hat{\theta }_r) > 1$ provides evidence for $\mathcal {H}_{0}$, whereas a Bayes factor ${{\,\textrm{BF}\,}}_{01}(\hat{\theta }_r) < 1$ provides evidence for $\mathcal {H}_{1}$. The more the Bayes factor deviates from one, the larger the evidence. In the following we will examine the Bayes factors related to various hypotheses about $\theta$ and $\alpha$.

#### Hypotheses about the effect size $\varvec{\theta }$

Researchers may be interested in testing the null hypothesis that there is no effect ($\mathcal {H}_{0} :\theta = 0$) against the alternative that there is an effect ($\mathcal {H}_{1} :\theta \ne 0$). We note that while the point null hypothesis $\mathcal {H}_{0}$ is often unrealistic, it is usually a good approximation to more realistic interval null hypotheses that assign a distribution tightly concentrated around zero (Berger and Delampady 1987; Ly and Wagenmakers 2022). Under $\mathcal {H}_{0}$ there are no free parameters, but under the alternative $\mathcal {H}_{1}$ the specification of a prior distribution for $\theta$ and $\alpha$ is required. A natural choice is to use the normalized power prior based on the original data along with a beta prior for the power parameter as in (2). The associated Bayes factor is then given by

7 $$\begin{aligned} {{\,\textrm{BF}\,}}_{01}\{\hat{\theta }_{r}\,\vert \,\mathcal {H}_{1} :\alpha \sim {{\,\textrm{Be}\,}}(x, y)\}&= \frac{f(\hat{\theta }_{r} \,\vert \,\mathcal {H}_{0} :\theta = 0)}{f\{\hat{\theta }_{r} \,\vert \,\mathcal {H}_{1} :\theta \,\vert \,\alpha \sim {{\,\textrm{N}\,}}(\hat{\theta }_{o}, \sigma ^{2}_{o}/\alpha ), \alpha \sim {{\,\textrm{Be}\,}}(x, y)\}} \nonumber \\&= \frac{{{\,\textrm{N}\,}}(\hat{\theta }_{r}\,\vert \,0, \sigma ^{2}_{r})}{\int _{0}^{1} {{\,\textrm{N}\,}}(\hat{\theta }_{r}\,\vert \,\hat{\theta }_{o}, \sigma ^{2}_{r} + \sigma ^{2}_{o}/\alpha ) \, {{\,\textrm{Be}\,}}(\alpha \,\vert \,x, y) \, \text {d}\alpha }. \end{aligned}$$

An intuitively reasonable choice for the prior of $\alpha$ under $\mathcal {H}_{1}$ is a uniform $\alpha \sim {{\,\textrm{Be}\,}}(x=1, y=1)$ distribution. However, it is worth noting that assigning a point mass $\alpha = 1$ leads to

8 $$\begin{aligned} {{\,\textrm{BF}\,}}_{01}(\hat{\theta }_{r}\,\vert \,\mathcal {H}_{1} :\alpha = 1)&= \frac{f(\hat{\theta }_{r} \,\vert \,\mathcal {H}_{0} :\theta = 0)}{f\{\hat{\theta }_{r} \,\vert \,\mathcal {H}_{1} :\theta \,\vert \,\alpha \sim {{\,\textrm{N}\,}}(\hat{\theta }_{o}, \sigma ^{2}_{o}/\alpha ), \alpha = 1\}} \nonumber \\&= \frac{{{\,\textrm{N}\,}}(\hat{\theta }_{r}\,\vert \,0, \sigma ^{2}_{r})}{ {{\,\textrm{N}\,}}(\hat{\theta }_{r}\,\vert \,\hat{\theta }_{o}, \sigma ^{2}_{o} + \sigma ^{2}_{r})}, \end{aligned}$$

which is the *replication Bayes factor* under normality (Verhagen and Wagenmakers 2014; Ly et al. 2018; Pawel and Held 2022), that is, the Bayes factor contrasting a point null hypothesis to the posterior distribution of the effect size based on the original data (and in this case a uniform initial prior). A fixed $\alpha = 1$ can also be seen as the limiting case of a beta prior with $y > 0$ and $x \rightarrow \infty$. The power prior version of the replication Bayes factor is thus a generalization of the standard replication Bayes factor, one that allows the original data to be discounted to some degree.

#### Hypotheses about the power parameter $\varvec{\alpha }$

To quantify the compatibility between the original and replication study, researchers may also be interested in testing hypotheses regarding the power parameter $\alpha$. For example, we may want to test the hypothesis that the data sets are “compatible” and should be completely pooled ($\mathcal {H}_{\text {c}} :\alpha = 1$) against the hypothesis that they are incompatible or “different” and the original data should be discounted to some extent ($\mathcal {H}_{\text {d}} :\alpha < 1$).

One approach is to assign a point prior $\mathcal {H}_{\text {d}}:\alpha = 0$ which represents the extreme position that the original data should be completely discounted. This leads to the issue that for a flat initial prior $f(\theta ) \propto 1$, the power prior with $\alpha = 0$ is not proper and so the resulting Bayes factor is only defined up to an arbitrary constant. Instead of the flat prior, we may thus assign an uninformative but proper initial prior to $\theta$, for instance, a unit-information prior $\theta \sim {{\,\textrm{N}\,}}(0, \kappa ^{2})$ with $\kappa ^{2}$ the variance from one (effective) observation (Kass and Wasserman 1995) as it encodes minimal prior information about the direction or magnitude of the effect size (Best et al. 2021). Updating the unit-information prior by the likelihood of the original data raised to the power of $\alpha$ leads then to a $\theta \,\vert \,\alpha \sim {{\,\textrm{N}\,}}\{\mu _{\alpha } = (\alpha \hat{\theta }_{o})/(\alpha + \sigma ^2_{o}/\kappa ^{2}), \sigma ^{2}_{\alpha } = 1/(1/\kappa ^{2} + \alpha /\sigma ^{2}_{o})\}$ distribution, so the Bayes factor is

9 $$\begin{aligned} {{\,\textrm{BF}\,}}_{\text {dc}}(\hat{\theta }_{r} \,\vert \,\mathcal {H}_{\text {d}}:\alpha = 0)&= \frac{f\{\hat{\theta }_{r} \,\vert \,\mathcal {H}_{\text {d}}:\theta \,\vert \,\alpha \sim {{\,\textrm{N}\,}}(\mu _{\alpha }, \sigma ^{2}_{\alpha }), \alpha = 0\}}{f\{\hat{\theta }_{r} \,\vert \,\mathcal {H}_{\text {c}}:\theta \,\vert \,\alpha \sim {{\,\textrm{N}\,}}(\mu _{\alpha }, \sigma ^{2}_{\alpha }), \alpha = 1\}}\nonumber \\&= \frac{{{\,\textrm{N}\,}}(\hat{\theta }_{r}\,\vert \,0, \sigma ^{2}_{r} + \kappa ^{2})}{{{\,\textrm{N}\,}}(\hat{\theta }_{r}\,\vert \,s \hat{\theta }_{o}, \sigma ^{2}_{r} + s \sigma ^{2}_{o})} \end{aligned}$$

with $$s = 1 / (1 + \sigma ^{2}_{o}/\kappa ^{2})$$.

An alternative approach that avoids the specification of a proper initial prior for $\theta$ is to assign a prior to $\alpha$ under $\mathcal {H}_{\text {d}}$. A suitable class of priors is given by $\mathcal {H}_{\text {d}} :\alpha \sim {{\,\textrm{Be}\,}}(1, y)$ with $y > 1$. The ${{\,\textrm{Be}\,}}(1, y)$ prior has its highest density at $\alpha = 0$ and is monotonically decreasing thus representing the more nuanced position that the original data should only be partially discounted. The parameter *y* determines the extent of partial discounting and the simple hypothesis $\mathcal {H}_{\text {d}} :\alpha = 0$ can be seen as a limiting case when $y \rightarrow \infty$. The resulting Bayes factor is given by

10 $$\begin{aligned} {{\,\textrm{BF}\,}}_{\text {dc}}\{\hat{\theta }_{r}\,\vert \,\mathcal {H}_{\text {d}} :\alpha \sim {{\,\textrm{Be}\,}}(1, y)\}&= \frac{f\{\hat{\theta }_{r} \,\vert \,\mathcal {H}_{\text {d}}:\theta \,\vert \,\alpha \sim {{\,\textrm{N}\,}}(\hat{\theta }_{o}, \sigma ^{2}_{o}/\alpha ), \alpha \sim {{\,\textrm{Be}\,}}(1, y)\}}{f\{\hat{\theta }_{r} \,\vert \,\mathcal {H}_{\text {c}} :\theta \,\vert \,\alpha \sim {{\,\textrm{N}\,}}(\hat{\theta }_{o}, \sigma ^{2}_{o}/\alpha ), \alpha = 1\}} \nonumber \\&= \frac{\int _{0}^{1}{{\,\textrm{N}\,}}(\hat{\theta }_{r}\,\vert \,\hat{\theta }_{o}, \sigma ^{2}_{r} + \sigma ^{2}_{o}/\alpha ) \, {{\,\textrm{Be}\,}}(\alpha \,\vert \,1, y) \, \text {d}\alpha }{{{\,\textrm{N}\,}}(\hat{\theta }_{r}\,\vert \,\hat{\theta }_{o}, \sigma ^{2}_{r} + \sigma ^{2}_{o})}. \end{aligned}$$

#### Example “Labels” (continued)

Table 1 displays the results of the proposed hypothesis tests applied to the three replications of the “Labels” experiment. The Bayes factors contrasting $\mathcal {H}_{0}:\theta = 0$ to $\mathcal {H}_{1}:\theta \ne 0$ with normalized power prior with uniform prior for the power parameter $\alpha$ under the alternative (column ${{\,\textrm{BF}\,}}_{01}\{{\hat{\theta }}_r \,\vert \,\mathcal {H}_{1} :\alpha \sim {{\,\textrm{Be}\,}}(1, 1)\}$) indicate neither evidence for absence nor presence of an effect in the first replication, but decisive evidence for the presence of an effect in the second and third replication. In all three cases, the Bayes factors are close to the standard replication Bayes factors with $\alpha = 1$ under the alternative (column ${{\,\textrm{BF}\,}}_{01}({\hat{\theta }}_r \,\vert \,\mathcal {H}_{1} :\alpha = 1)$).

**Table 1:** Hypothesis tests for the replication studies of the “Labels” experiment with original standardized mean difference effect estimate $\hat{\theta }_{o} = 0.21$ and standard error $\sigma _{o} = 0.05$

	${\hat{\theta }}_r$	$\sigma _r$	Tests about the effect size $\theta$		Tests about the power parameter $\alpha$	
			${{\,\textrm{BF}\,}}_{01}\{{\hat{\theta }}_r \,\vert \,\mathcal {H}_{1} :\alpha \sim {{\,\textrm{Be}\,}}(1, 1)\}$	${{\,\textrm{BF}\,}}_{01}({\hat{\theta }}_r \,\vert \,\mathcal {H}_{1} :\alpha = 1)$	${{\,\textrm{BF}\,}}_{\text {dc}}({\hat{\theta }}_r \,\vert \,\mathcal {H}_{\text {d}} :\alpha = 0)$	${{\,\textrm{BF}\,}}_{\text {dc}}\{{\hat{\theta }}_r \,\vert \,\mathcal {H}_{\text {d}} :\alpha \sim {{\,\textrm{Be}\,}}(1, 2)\}$
1	0.09	0.05	1/1.1	1.1	1/5.6	1.2
2	0.21	0.06	1/367	1/478	1/19	1/1.5
3	0.44	0.04	< 1/1000	< 1/1000	16	25

The columns indicate replication effect estimates $\hat{\theta }_{r}$, their standard errors $\sigma _{r}$, Bayes factors contrasting the absence of an effect $\mathcal {H}_{0}:\theta = 0$ to the presence of an effect $\mathcal {H}_{1} :\theta \ne 0$ with either a uniform prior $\alpha \sim {{\,\textrm{Be}\,}}(x = 1, y = 1)$ or point prior $\alpha = 1$ under $\mathcal {H}_{1}$, and Bayes factors contrasting study incompatibility $\mathcal {H}_{\text {d}}:\alpha < 1$ to study compatibility $\mathcal {H}_{\text {c}}:\alpha = 1$ with either complete discounting prior $\alpha = 0$ or partial discounting prior $\alpha \sim {{\,\textrm{Be}\,}}(1, y = 2)$ under $\mathcal {H}_{\text {d}}$

In order to compute the Bayes factor for testing $\mathcal {H}_{\text {d}}:\alpha = 0$ versus $\mathcal {H}_{\text {c}}:\alpha = 1$ we need to specify a unit variance for the unit-information prior. A crude approximation for the variance of a standardized mean difference effect estimate is given by ${{\,\textrm{Var}\,}}(\hat{\theta }_i) = 4/n_{i}$ with $n_{i}$ the total sample size of the study, and assuming equal sample size in both groups (Hedges and Schauer 2021, p. 5). We may thus set the variance of the unit-information prior to $\kappa ^{2} = 2$ since a total sample size of $n_{i} = 2$ (at least one observation from each group) is required to estimate a standardized mean difference. Based on this choice, the Bayes factors ${{\,\textrm{BF}\,}}_{\text {dc}}({\hat{\theta }}_r \,\vert \,\mathcal {H}_{\text {d}}:\alpha = 0)$ in Table 1 indicate that the data provide substantial and strong evidence for the compatibility hypothesis $\mathcal {H}_{\text {c}}$ in the first and second replication study, respectively, whereas the data indicate strong evidence for complete incompatibility $\mathcal {H}_{\text {d}}$ in the third replication study. The Bayes factor ${{\,\textrm{BF}\,}}_{\text {dc}}\{{\hat{\theta }}_r \,\vert \,\mathcal {H}_{\text {d}}:\alpha \sim {{\,\textrm{Be}\,}}(1, y = 2)\}$ in the right-most column with the partial discounting prior assigned under hypothesis $\mathcal {H}_{\text {d}}$ indicates absence of evidence for either hypothesis in the first and second replication, but strong evidence for incompatibility $\mathcal {H}_{\text {d}}$ in the third replication. The apparent differences to the Bayes factor with the complete discounting prior (column ${{\,\textrm{BF}\,}}_{\text {dc}}({\hat{\theta }}_r \,\vert \,\mathcal {H}_{\text {d}}:\alpha = 0)$) illustrate that in case of no conflict (study 2) or not too much conflict (study 1) the test with the partial discounting prior is less sensitive in diagnosing (in)compatibility, but in case of substantial conflict (study 3) it is more sensitive.

**Fig. 2:**
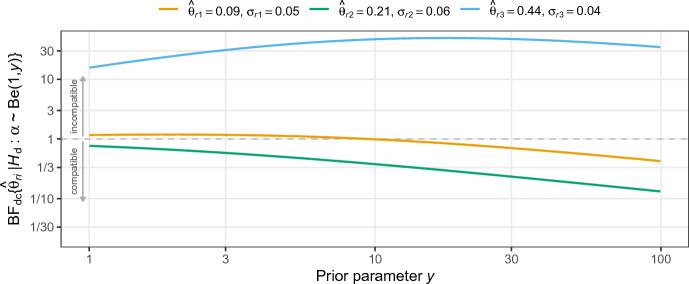
Sensitivity of the Bayes factor ${{\,\textrm{BF}\,}}_{\text {dc}}\{{\hat{\theta }}_r \,\vert \,\mathcal {H}_{\text {d}}:\alpha \sim {{\,\textrm{Be}\,}}(1, y)\}$ with respect to the parameter *y* of the partial discounting prior under $\mathcal {H}_{\text {d}}$

The previous analysis is based on a beta prior with $y = 2$ corresponding to a linearly decreasing density in $\alpha$, Fig. 2 shows the Bayes factor for other values of *y*. We see that in the realistic range of $y = 1$ (uniform prior) to $y = 100$ (almost all mass at $\alpha = 0$) the results for the first and third replication hardly change, while for the second replication the Bayes factor shifts from anecdotal evidence to stronger evidence for compatibility.

To conclude, our analysis suggests that only the second replication was fully successful in the sense that it provides evidence for the presence of an effect while also being compatible with the original study. For the other two replications the conclusions are more nuanced: In the first replication, there is neither evidence for the absence nor the presence of an effect, but substantial evidence for compatibility when a complete discounting prior is used, and no evidence for (in)compatibility when a partial discounting prior is used. Finally, in the third replication there is decisive evidence for an effect, but also strong evidence of incompatibility with the original study.

#### Bayes factor asymptotics

Some of the Bayes factors in the previous example provided only modest evidence for the test-relevant hypotheses despite the large sample sizes in original and replication study. It is therefore of interest to understand the asymptotic behavior of the proposed Bayes factors. For instance, we may wish to understand what happens when the standard error of the replication study $\sigma _{r}$ becomes arbitrarily small (through an increase in sample size). Assume that $\hat{\theta }_{r}$ is a consistent estimator of its true underlying effect size $\theta _r$, so that as the standard error $\sigma _r$ goes to zero, the estimate will converge in probability to the true effect size $\theta _r$. The true replication effect size $\theta _{r}$ may be different from the true original effect size $\theta _{o}$, for example, because the participant populations from both studies systematically differ.

The limiting Bayes factors for testing the effect size $\theta$ from (7) and (8) are then given by

$$\begin{aligned} \lim _{\sigma _{r} \downarrow 0} {{\,\textrm{BF}\,}}_{\text {01}} \{\hat{\theta }_r \,\vert \,\mathcal {H}_{1} :\alpha \sim {{\,\textrm{Be}\,}}(x, y)\}&= \frac{\delta (\theta _r) \, \sqrt{2\pi } \, \text{ B }(x, y)}{\text{ B }(x + 1/2, y)} \, \\&\quad \times M\bigg \{x+1/2, x + y + 1/2, -\frac{(\theta _r - \hat{\theta }_o)^2}{2\sigma ^2_o}\bigg \}^{-1} \end{aligned}$$

and

$$\begin{aligned} \lim _{\sigma _{r} \downarrow 0} {{\,\textrm{BF}\,}}_{\text {01}} (\hat{\theta }_r \,\vert \,\mathcal {H}_{1} :\alpha = 1)&= \frac{\delta (\theta _r)}{{{\,\textrm{N}\,}}(\theta _r \,\vert \,\hat{\theta }_o, \sigma ^2_o)}, \end{aligned}$$

with $\delta (\cdot )$ the Dirac delta function. Both Bayes factors are hence consistent (Bayarri et al. 2012) in the sense that they indicate overwhelming evidence for the correct hypothesis (i. e.,  the Bayes factors go to infinity/zero if the true effect size $\theta _r$ is zero/non-zero). In contrast, the Bayes factors for testing the power parameter $\alpha$ from (9) and (10) converge to positive constants

11 $$\begin{aligned} \lim _{\sigma _{r} \downarrow 0} {{\,\textrm{BF}\,}}_{\text {dc}} (\theta _r\,\vert \,\mathcal {H}_{\text {d}} :\alpha = 0) = \sqrt{1 - s} \, \exp \left[ -\frac{1}{2} \, \left\{ \frac{\theta _r^{2}}{\kappa ^{2}} - \frac{(\theta _r - s\hat{\theta }_{o})^{2}}{s\sigma ^{2}_{o}}\right\} \right] \end{aligned}$$

and

12 $$\begin{aligned} \lim _{\sigma _{r} \downarrow 0} {{\,\textrm{BF}\,}}_{\text {dc}}\{\theta _r\,\vert \,\mathcal {H}_{\text {d}} :\alpha \sim {{\,\textrm{Be}\,}}(1, y)\}&= \frac{\text{ B }(3/2, y)}{\text{ B }(1, y)} \, M\left\{ y, y + 3/2, \frac{(\theta _r - \hat{\theta }_o)^2}{2\sigma ^2_o}\right\} . \end{aligned}$$

The amount of evidence one can find for either hypothesis thus depends on the original effect estimate $\hat{\theta }_{o}$, the standard error $\sigma _{o}$, and the true effect size $\theta _r$. For instance, in the “Labels” experiment we have an original effect estimate $\hat{\theta }_{o} = 0.21$, a standard error $\sigma _{o} = 0.05$, and a unit variance $\kappa ^{2}=2$. The bound (11) is minimized for a true effect size equal to the original effect estimate $\theta _r = \hat{\theta }_{o} = 0.21$, so the most extreme level we can obtain is $\lim _{\sigma _{r} \downarrow 0} {{\,\textrm{BF}\,}}_{\text {dc}} (\theta _r\,\vert \,\mathcal {H}_{\text {d}} :\alpha = 0) = 1/28$. Similarly, the bound (12) is minimized for $\theta _r = \hat{\theta }_{o} = 0.21$ since then the confluent hypergeometric function term becomes one, leading to $\lim _{\sigma _{r} \downarrow 0} {{\,\textrm{BF}\,}}_{\text {dc}} \{\theta _r\,\vert \,\mathcal {H}_{\text {d}} :\alpha \sim {{\,\textrm{Be}\,}}(1, y = 2)\} = \text{ B }(3/2, y)/\text{B }(1,y) = 1/1.9$. Even in a perfectly precise replication study we cannot find more evidence, and hence the posterior probability of $\mathcal {H}_{\text {c}}:\alpha = 1$ cannot converge to one.

While the Bayes factors (9) and (10) are inconsistent if the replication data become arbitrarily informative, the situation is different when also the original data become arbitrarily informative (reflected by also the standard error $\sigma _o$ going to zero and the original effect estimate $\hat{\theta }_o$ converging to its true effect size $\theta _o$). The Bayes factor with $\mathcal {H}_{\text {d}}:\alpha = 0$ from (9) is then consistent as the limit (11) goes correctly to infinity/zero if the true effect size of the replication study $\theta _r$ is different/equivalent from the true effect size of the original study $\theta _o$. In contrast, the Bayes factor with $\mathcal {H}_{\text {d}} :\alpha \sim {{\,\textrm{Be}\,}}(1, y)$ from (10) is still inconsistent since it only shows the correct asymptotic behavior when the true effect sizes are unequal (i. e.,  the Bayes factor goes to infinity) but not when the effect sizes are equivalent, in which case it is still bounded by $\text{ B }(3/2, y)/\text{B }(1,y)$.

#### Bayes factor design of replication studies

Now assume that the replication study has not yet been conducted and we wish to plan for a suitable sample size. The design of replication studies should be aligned with the planned analysis (Anderson and Maxwell 2017) and if multiple analyses are performed, a sample size may be calculated that guarantees a sufficiently conclusive analysis in each case (Pawel et al. 2023b). In the power prior framework, samples size calculations may be based on either hypothesis testing or estimation of the effect size $\theta$ or the power parameter $\alpha$. Estimation based approaches have been developed by Shen et al. (2023). Here, we focus on samples size calculations based on Bayes factor hypothesis testing as the methodology is still lacking.

In the case of testing the effect size $\theta$, Pawel and Held (2022) studied Bayesian design of replication studies based on the Bayes factor (8) with $\alpha = 1$ under $\mathcal {H}_{1}$, i.e., the replication Bayes factor under normality. They obtained closed-form expressions for the probability of replication success under $\mathcal {H}_{0}$ and $\mathcal {H}_{1}$ based on which standard Bayesian design can be performed (Weiss 1997; Gelfand and Wang 2002; De Santis 2004; Schönbrodt and Wagenmakers 2017). For the Bayes factor (7) with $\alpha \sim {{\,\textrm{Be}\,}}(x, y )$ under $\mathcal {H}_{1}$, closed-form expressions are not available anymore and simulation or numerical integration have to be used for sample size calculations.

For tests related to the power parameter $\alpha$, there are also closed-form expressions for the probability of replication success based on the Bayes factor (9) with $\alpha = 0$ under $\mathcal {H}_{\text {d}}$. We will now show how these can be derived and used for determining the replication sample size. With some algebra, one can show that ${{\,\textrm{BF}\,}}_{\text {dc}}(\hat{\theta }_{r} \,\vert \,\mathcal {H}_{\text {d}}:\alpha = 0) \le \gamma$ is equivalent to

13 $$\begin{aligned} \left\{ \hat{\theta }_{r} - \frac{\hat{\theta }_o \, (\sigma ^{2}_{r} + \kappa ^{2})}{\kappa ^2}\right\} ^{2} \le X \end{aligned}$$

with

$$\begin{aligned} X =&\frac{(\sigma ^{2}_{r} + \kappa ^{2})(\sigma ^{2}_{r} + s \sigma ^{2}_{o})}{\kappa ^{2} - s \sigma ^{2}_{o}} \left\{ \log \gamma ^{2}- \log \left( \frac{\sigma ^{2}_{r} + s \sigma ^{2}_{o}}{ \sigma ^{2}_{r} + \kappa ^{2}}\right) - \frac{s^{2} \hat{\theta }^{2}_{o}}{s \sigma ^{2}_{o} - \kappa ^{2}}\right\} \end{aligned}$$

and $s = 1 / (1 + \sigma ^{2}_{o}/\kappa ^{2})$. Denote by $m_{i}$ and $v_{i}$ the mean and variance of $\hat{\theta }_{r}$ under hypothesis $i \in \{\text {d}, \text {c}\}$. The left hand side of (13) then follows a scaled non-central chi-squared distribution under both hypotheses. Hence the probability of replication success is given by

14 $$\begin{aligned} \Pr ({{\,\textrm{BF}\,}}_{\text {dc}} \le \gamma \,\vert \,\mathcal {H}_{i})&= \Pr \left( \chi ^{2}_{1,\lambda _{i}} \le X/v_{i} \right) \end{aligned}$$

with non-centrality parameter

$$\begin{aligned} \lambda _{i} = \left\{ m_{i} - \frac{\hat{\theta }_o \, (\sigma ^{2}_{r} + \kappa ^{2})}{\kappa ^2} \right\} ^{2} \big / v_{i}. \end{aligned}$$

To determine the replication sample size, we can now use (14) to compute the probability of replication success at a desired level $\gamma$ over a grid of replication standard errors $\sigma _{r}$, and under either hypothesis $\mathcal {H}_{\text {d}}$ and $\mathcal {H}_{\text {c}}$. The appropriate standard error $\sigma _r$ is then chosen so that the probability for finding correct evidence is sufficiently high under the respective hypothesis, and sufficiently low under the wrong hypothesis. Subsequently, the standard error $\sigma _{r}$ needs to be translated into a sample size, e. g.,  for standardized mean differences via the aforementioned approximation $n_{r} \approx 4/\sigma ^{2}_{r}$.

**Fig. 3:**
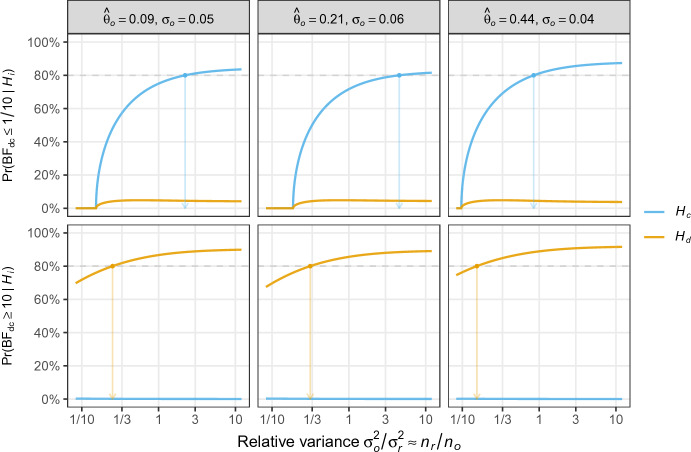
Probability of replication success as a function of relative variance for the three replications of experiment “Labels” regarded as original study. The arrows point to the relative variance associated with an 80% probability under the respective hypotheses

#### Example “Labels” (continued)

Figure 3 illustrates Bayesian design based on the Bayes factor ${{\,\textrm{BF}\,}}_{\text {dc}}(\hat{\theta }_{r} \,\vert \,\mathcal {H}_{\text {d}} :\alpha = 0)$ testing the power parameter $\alpha$ from (9). The three replication studies from the experiment “Labels” are now regarded as original studies, and each column of the figure shows the corresponding design of future replications. In each plot, the probability for finding strong evidence for $\mathcal {H}_{\text {c}}:\alpha = 1$ (top) or $\mathcal {H}_{\text {d}}:\alpha = 0$ (bottom) is shown as a function of the relative sample size. In both cases, the probability is computed assuming that either $\mathcal {H}_{\text {c}}$ (blue) or $\mathcal {H}_{\text {d}}$ (yellow) is true.

The curves look more or less similar for all three studies. We see from the lower panels that the probability for finding strong evidence for $\mathcal {H}_{\text {d}}$ is not much affected by the sample size of the replication study; it stays at almost zero under $\mathcal {H}_{\text {c}}$, while under $\mathcal {H}_{\text {d}}$ it increases from about 75% to about 90%. In contrast, the top panels show that the probability for finding strong evidence for $\mathcal {H}_{\text {c}}$ rapidly increases under $\mathcal {H}_{\text {c}}$ and seems to level off at an asymptote. Under $\mathcal {H}_{\text {d}}$ the probability stays below 5% across the whole range.

The arrows in the plots also display the required relative sample size to obtain strong evidence with probability of $80\%$ under the correct hypothesis. We see that original studies with smaller standard errors require smaller relative sample sizes in the replication to achieve the same probability of replication success. Under $\mathcal {H}_{\text {c}}$ the required relative sample sizes are larger than under $\mathcal {H}_{\text {d}}$. However, while the probability of misleading evidence under $\mathcal {H}_{\text {c}}$ seems to be well controlled under the determined sample size, under $\mathcal {H}_{\text {d}}$ it stays roughly 5% for all three studies, and even for very large replication sample sizes. Choosing the sample size based on finding strong evidence for $\mathcal {H}_{\text {c}}$ assuming $\mathcal {H}_{\text {c}}$ is true thus also guarantees appropriate error probabilities for finding strong evidence for $\mathcal {H}_{\text {d}}$ in all three studies. At the same time, it seems that the probability for finding misleading evidence for $\mathcal {H}_{\text {c}}$ cannot be reduced below around 5% which might be undesirably high for certain applications.

## Connection to hierarchical modeling of replication studies

Hierarchical modeling is another approach that allows for the incorporation of historical data in Bayesian analyses; moreover, hierarchical models have previously been used in the replication setting (Bayarri and Mayoral 2002a, b; Pawel and Held 2020). We will now investigate how the hierarchical modeling approach is related to the power prior approach in the analysis of replication studies, both in parameter estimation and hypothesis testing.

### Connection to parameter estimation in hierarchical models

Assume a hierarchical model

15a $$\begin{aligned} \hat{\theta }_{i} \,\vert \,\theta _{i}\,&\sim {{\,\textrm{N}\,}}(\theta _{i}, \sigma ^{2}_{i}) \end{aligned}$$

15b $$\begin{aligned} \theta _{i} \,\vert \,\theta _{*}&\sim {{\,\textrm{N}\,}}(\theta _{*}, \tau ^{2}) \end{aligned}$$

15c $$\begin{aligned} f(\theta _{*})&\propto k \end{aligned}$$

where for study $i \in \{o,r\}$ the effect estimate $\hat{\theta }_i$ is normally distributed around a study specific effect size $\theta _i$ which itself is normally distributed around an overall effect size $\theta _{*}$. The heterogeneity variance $\tau ^2$ determines the similarity of the study specific effect sizes $\theta _i$. The overall effect size $\theta _*$ is assigned an (improper) flat prior $f(\theta _{*}) \propto k$, for some $k > 0$, which is a common approach in hierarchical modeling of effect estimates (Röver et al. 2021).

We show in Appendix A that under the hierarchical model (15) the marginal posterior distribution of the replication specific effect size $\theta _{r}$ is given by

16 $$\begin{aligned} \theta _{r} \,\vert \,\hat{\theta }_{o}, \hat{\theta }_{r}, \tau ^{2} \sim {{\,\textrm{N}\,}}\left( \frac{\hat{\theta }_{r}/\sigma ^{2}_{r} + \hat{\theta }_{o}/(2\tau ^{2} + \sigma ^{2}_{o})}{1/\sigma ^{2}_{r} + 1/(2\tau ^{2} + \sigma ^{2}_{o})}, \frac{1}{1/\sigma ^{2}_{r} + 1/(2\tau ^{2} + \sigma ^{2}_{o})}\right) , \end{aligned}$$

that is, a normal distribution whose mean is a weighted average of the replication effect estimate $\hat{\theta }_r$ and the original effect estimate $\hat{\theta }_o$. The amount of shrinkage of the replication towards the original effect estimate depends on how large the replication standard error $\sigma _r$ is relative to the heterogeneity variance $\tau ^2$ and the original standard error $\sigma _o$. There exists a correspondence between the posterior for the replication effect size $\theta _r$ from the hierarchical model (16) and the posterior for the effect size $\theta$ under the power prior approach. Specifically, note that under the power prior and for a fixed power parameter $\alpha$, the posterior of the effect size $\theta$ is given by

17 $$\begin{aligned} \theta \,\vert \,\hat{\theta }_{o}, \hat{\theta }_{r}, \alpha \sim {{\,\textrm{N}\,}}\left( \frac{\hat{\theta }_{r}/\sigma ^{2}_{r} + (\hat{\theta }_{o}\alpha )/\sigma ^{2}_{o}}{1/\sigma ^{2}_{r} + \alpha /\sigma ^{2}_{o}}, \frac{1}{1/\sigma ^{2}_{r} + \alpha /\sigma ^{2}_{o}}\right) . \end{aligned}$$

**Fig. 4:**
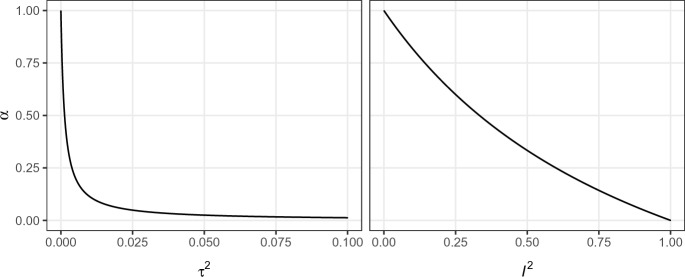
The heterogeneity $\tau ^{2}$ and relative heterogeneity $I^{2} = \tau ^{2}/(\tau ^{2} + \sigma ^{2}_o)$ of a hierarchical model versus the power parameter $\alpha$ from a power prior model which lead to matching posteriors for the effect sizes $\theta$ and $\theta _{r}$. The variance of the original effect estimate $\sigma _{o}^2 = 0.05^{2}$ from the “Labels” experiment is used for the transformation to the heterogeneity scale $\tau ^{2}$

The hierarchical posterior  (16) and the power prior posterior (17) thus match if and only if

18 $$\begin{aligned} \alpha = \frac{\sigma ^{2}_{o}}{2\tau ^{2} + \sigma ^{2}_{o}}, \end{aligned}$$

respectively

19 $$\begin{aligned} \tau ^{2} = \left( \frac{1}{\alpha } - 1\right) \, \frac{\sigma ^{2}_{o}}{2}, \end{aligned}$$

which was first shown by Chen and Ibrahim (2006). For instance, a power prior model with $\alpha = 1$ corresponds to a hierarchical model with $\tau ^{2} = 0$, and a hierarchical model with $\tau ^2 \rightarrow \infty$ corresponds to a power prior model with $\alpha \downarrow 0$. In between these two extremes, however, $\alpha$ has to be interpreted as a relative measure of heterogeneity since the transformation to $\tau ^2$ involves a scaling by the variance $\sigma ^2_o$ of the original effect estimate. For this reason, there is a direct correspondence between $\alpha$ and the popular relative heterogeneity measure $I^{2} = \tau ^{2}/(\tau ^{2} + \sigma ^{2}_o)$ (Higgins and Thompson 2002) computed from $\tau ^2$ and the variance of the original estimate $\sigma ^2_o$, that is,

$$\begin{aligned} \alpha = \frac{1 - I^{2}}{1 + I^{2}}, \end{aligned}$$

with inverse of the same functional form. Figure 4 shows $\alpha$ and the corresponding $\tau ^2$ and $I^2$ values which lead to matching posteriors.

It has remained unclear whether or not a similar correspondence exists in cases where $\alpha$ and $\tau ^{2}$ are random and assigned prior distributions. Here we confirm that there is indeed such a correspondence. Specifically, the marginal posterior of the replication effect size $\theta _r$ from the hierarchical model matches with the marginal posterior of the effect size $\theta$ from the power prior model if the prior density functions $f_{\tau ^2}(\cdot )$ and $f_{\alpha }(\cdot )$ of $\tau ^2$ and $\alpha$ satisfy

20 $$\begin{aligned} f_{\tau ^2}(\tau ^{2}) = f_{\alpha }\left( \frac{\sigma ^{2}_{o}}{2 \tau ^{2} + \sigma ^{2}_{o}}\right) \, \frac{2\sigma ^{2}_{o}}{(2 \tau ^{2} + \sigma ^{2}_{o})^{2}} \end{aligned}$$

for every $\tau ^2 \ge 0$, see Appendix B for details. Importantly, the correspondence condition (20) involves a scaling by the variance from the original effect estimate $\sigma ^2_o$, meaning that also in this case $\alpha$ acts similar to a relative heterogeneity parameter. This can also be seen from the correspondence condition between $\alpha$ and $I^2 = \tau ^2/(\sigma ^2_o + \tau ^2)$, which can be derived in exactly the same way as the correspondence between $\alpha$ and $\tau ^2$. That is, the marginal posteriors of $\theta$ and $\theta _r$ match if the prior density functions $f_{I^2}(\cdot )$ and $f_{\alpha }(\cdot )$ of $I^2$ and $\alpha$ satisfy

21 $$\begin{aligned} f_{I^2}(I^{2}) = f_{\alpha }\left( \frac{1 - I^2}{1 + I^2}\right) \frac{2}{(1 + I^2)^2} \end{aligned}$$

for every $$0 \le I^2 \le 1$$.

Interestingly, conditions (21) and (20) imply that a beta prior on the power parameter $\alpha \sim {{\,\textrm{Be}\,}}(x, y)$ corresponds to a generalized F prior on the heterogeneity $\tau ^2 \sim \text{ GF }(y, x, 2/\sigma ^2_o)$ and a generalized beta prior on the relative heterogeneity $I^2 \sim \text{ GBe }(y, x, 2)$, see Appendix C for details on both distributions. This connection provides a convenient analytical link between hierarchical modeling and the power prior framework, as beta priors for $\alpha$ are almost universally used in applications of power priors. The result also illustrates that the power prior framework seems unnatural from the perspective of hierarchical modeling since it corresponds to specifying priors on the $I^2$ scale rather than on the $\tau ^2$ scale. The same prior on $I^2$ will imply different degrees of informativeness on the $\tau ^2$ scale for original effect estimates $\hat{\theta }_o$ with different variances $\sigma ^2_o$ since $I^2$ is entangled with the variance of the original effect estimate.

**Fig. 5:**
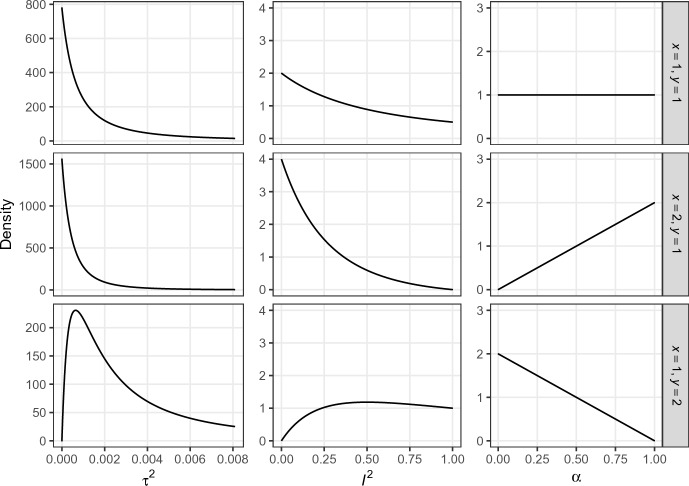
Priors on the heterogeneity $\tau ^2 \sim \text{ GF }(y, x, 2/\sigma ^2_o)$ (left), the relative heterogeneity $I^2 = \tau ^2/(\sigma ^2_o + \tau ^2) \sim \text{ GBe }(y, x, 2)$ (middle) and the power parameter $\alpha \sim {{\,\textrm{Be}\,}}(x, y)$ (right) that lead to matching marginal posteriors for effect sizes $\theta$ and $\theta _{r}$. The variance of the original effect estimate $\sigma _{o}^2 = 0.05^{2}$ from the “Labels” experiment is used for the transformation to the heterogeneity scale $\tau ^{2}$

Figure 5 provides three examples of matching priors using the variance of the original effect estimate from the “Labels” experiment for the transformation to the heterogeneity scale $\tau ^2$. The top row of Fig. 5 shows that the uniform prior on $\alpha$ corresponds to a $f(\tau ^{2}) \propto \sigma ^{2}_{o}/(2\tau ^{2} + \sigma ^{2}_{o})^{2}$ prior which is similar to the “uniform shrinkage” prior $f(\tau ^{2}) \propto \sigma ^{2}_{o}/(\tau ^{2} + \sigma ^{2}_{o})^{2}$ (Daniels 1999). This prior has the highest density at $\tau ^{2} = 0$ but still gives some mass to larger values of $\tau ^{2}$. Similarly, on the scale of $I^2$ the prior slightly favors smaller values. The middle row of Fig. 5 shows that the $\alpha \sim {{\,\textrm{Be}\,}}(2, 1)$ prior—indicating more compatibility between original and replication than the uniform prior—gives even more mass to small values of $\tau ^{2}$ and $I^2$, and also has the highest density at $\tau ^2 = 0$ and $I^2 = 0$. In contrast, the bottom row of Fig. 5 shows that the $\alpha \sim {{\,\textrm{Be}\,}}(1, 2)$ prior—indicating less compatibility between original and replication than the uniform prior—gives less mass to small $\tau ^{2}$ and $I^2$, and has zero density at $\tau ^{2} = 0$ and $I^2 = 0$.

### Connection to hypothesis testing in hierarchical models

Two types of hypothesis tests can be distinguished in the hierarchical model; tests for the overall effect size $\theta _*$ and tests for the heterogeneity variance $\tau ^2$. In all cases, computations of marginal likelihoods of the form

22 $$\begin{aligned} f(\hat{\theta }_r \,\vert \,\mathcal {H}_{i}) = \int {{\,\textrm{N}\,}}(\hat{\theta }_r \,\vert \,\theta _*, \sigma ^2_r + \tau ^2) \, f(\theta _*, \tau ^2 \,\vert \,\mathcal {H}_{i}) \, \text {d}\theta _* \, \text {d}\tau ^2 \end{aligned}$$

with $i \in \{j, k\}$ are required for obtaining Bayes factors ${{\,\textrm{BF}\,}}_{jk}(\hat{\theta }_r){=}f(\hat{\theta }_r \,\vert \,\mathcal {H}_{j}){/}f(\hat{\theta }_r \,\vert \,\mathcal {H}_{k})$ which quantify the evidence that the replication data $\hat{\theta }_r$ provide for a hypothesis $\mathcal {H}_{k}$ over a competing hypothesis $\mathcal {H}_{j}$. Under each hypothesis a joint prior for $\tau ^2$ and $\theta _*$ needs to be assigned.

As with parameter estimation, it is of interest to investigate whether there is a correspondence with hypothesis tests from the power prior framework from Sect. 2.2. For two tests to match, one needs to assign priors to $\tau ^2$ and $\theta _*$, respectively, to $\alpha$ and $\theta$ so that the marginal likelihood (22) equals the marginal likelihood from the power prior model (6) under both test-relevant hypotheses.

Concerning the generalized replication Bayes factor from (7) testing $\mathcal {H}_{0} :\theta = 0$ versus $\mathcal {H}_{1} :\theta \ne 0$, one can show that it matches with the Bayes factor contrasting $\mathcal {H}_{0} :\theta _* = 0$ versus $\mathcal {H}_{1} :\theta _* \ne 0$ with

$$\begin{aligned} \mathcal {H}_{0}:\theta _{*}&= 0{} & {} \text {versus}&\mathcal {H}_{1}:\theta _{*} \,\vert \,\tau ^{2} \sim {{\,\textrm{N}\,}}(\hat{\theta }_{o}, \sigma ^{2}_{o} + \tau ^{2}) \\ \tau ^{2}&= 0{} & {} {}&\tau ^2 \sim \text{ GF }(y, x, \sigma ^2_o/2) \end{aligned}$$

for the replication data in in the hierarchical framework. The Bayes factor thus compares the likelihood of the replication data under the hypothesis $\mathcal {H}_{0}$ postulating that the global effect size $\theta _{*}$ is zero and that there is no effect size heterogeneity, relative to the likelihood of the data under the hypothesis $\mathcal {H}_{1}$ postulating that $\theta _{*}$ follows the posterior based on the original data and an initial flat prior for $\theta _{*}$ along with a generalized F prior on the heterogeneity $\tau ^2$. Setting the heterogeneity to $\tau ^2 = 0$ under $\mathcal {H}_{1}$ instead produces the replication Bayes factor under normality from (8).

The Bayes factor (9) that tests complete discouting $\mathcal {H}_{\text {d}}:\alpha = 0$ versus complete compatibility $\mathcal {H}_{\text {c}}:\alpha = 1$ can be obtained in the hierarchical framework by contrasting

$$\begin{aligned} \mathcal {H}_{\text {d}}:\theta _{*}&\sim {{\,\textrm{N}\,}}(0, \kappa ^{2}){} & {} \text {versus}&\mathcal {H}_{\text {c}} :\theta _{*}&\sim {{\,\textrm{N}\,}}(s\,\hat{\theta }_{o}, s\,\sigma ^{2}_{o}) \\ \tau ^{2}&= 0{} & {} {}&\tau ^{2}&= 0 \end{aligned}$$

with $s = 1 / (1 + \sigma ^{2}_{o}/\kappa ^{2})$. Hence, the Bayes factor compares the likelihood of the replication data under the initial unit-information prior relative to the likelihood of the replication data under the unit-information prior updated by the original data, assuming no heterogeneity under either hypothesis (so that the hierarchical model collapses to a fixed effects model). Although this particular test relates to the power parameter $\alpha$ in the power prior model, it is surprisingly unrelated to testing the heterogeneity variance $\tau ^2$ in the hierarchical model.

The Bayes factor (10) testing $\mathcal {H}_{\text {d}}:\alpha < 1$ versus $\mathcal {H}_{\text {c}}:\alpha = 1$ using the partial discounting prior $\mathcal {H}_{\text {d}} :\alpha \sim {{\,\textrm{Be}\,}}(1, y)$ corresponds to testing $\mathcal {H}_{\text {d}}:\tau ^2 > 0$ versus $\mathcal {H}_{\text {c}}:\tau ^2 = 0$ with priors

$$\begin{aligned} \mathcal {H}_{\text {d}}:\theta _{*} \,\vert \,\tau ^{2}&\sim {{\,\textrm{N}\,}}(\hat{\theta }_{o}, \sigma ^{2}_{o} + \tau ^{2})&\text {versus}{} & {} \mathcal {H}_{\text {c}} :\theta _{*} \,\vert \,\tau ^{2}&\sim {{\,\textrm{N}\,}}(\hat{\theta }_{o}, \sigma ^{2}_{o} + \tau ^{2}) \\ \tau ^2&\sim \text{ GF }(y, 1, \sigma ^2_o/2){} & {} {}&\tau ^{2}&= 0 \end{aligned}$$

The test for compatibility via the power parameter $\alpha$ is thus equivalent to a test for compatibility via the heterogeneity $\tau ^2$ (to which a generalized F prior is assigned) after updating of a flat prior for $\theta _*$ with the data from the original study.

### Bayes factor asymptotics in the hierarchical model

Like the original test of $\mathcal {H}_{\text {c}}:\alpha = 1$ versus $\mathcal {H}_{\text {d}}:\alpha \sim {{\,\textrm{Be}\,}}(1, y)$, the corresponding test of $\tau ^2$ is inconsistent in the sense that when the standard errors from both studies go to zero ($\sigma _o \downarrow 0$ and $\sigma _r \downarrow 0$) and their true effect sizes are equivalent ($\theta _o = \theta _r$), the Bayes factor ${{\,\textrm{BF}\,}}_{\text {dc}}$ does not go to zero (to indicate overwhelming evidence for $\mathcal {H}_{\text {c}}:\tau ^2 = 0$) but converges to a positive constant. It is, however, possible to construct a consistent test for $\mathcal {H}_{\text {c}}:\tau ^2 = 0$ when we assign a different prior to $\tau ^2$ under $\mathcal {H}_{\text {d}}:\tau ^2 > 0$. For instance, when we assign an inverse gamma prior $\mathcal {H}_{\text {d}} :\tau ^2 \sim \text{ IG }(q, r)$ with shape *q* and scale *r*, the Bayes factor is given by

$$\begin{aligned} {{\,\textrm{BF}\,}}_{\text {dc}}\{\hat{\theta }_r \,\vert \,\mathcal {H}_{\text {d}} :\tau ^2 \sim \text{ IG }(q, r)\} = \frac{\int {{\,\textrm{N}\,}}(\hat{\theta }_r \,\vert \,\hat{\theta }_o, \sigma ^2_r + \sigma ^2_o + 2\tau ^2) \, \text{ IG }(\tau ^2 \,\vert \,q, r) \, \text {d}\tau ^2}{{{\,\textrm{N}\,}}(\hat{\theta }_r \,\vert \,\hat{\theta }_o, \sigma ^2_r + \sigma ^2_o)} \end{aligned}$$

with $\text{ IG }(\cdot \,\vert \,q, r)$ the density function of the inverse gamma distribution. The limiting Bayes factor is therefore

$$\begin{aligned} \lim _{\sigma _o, \sigma _r \downarrow 0} {{\,\textrm{BF}\,}}_{\text {dc}}\{\hat{\theta }_r \,\vert \,\mathcal {H}_{\text {d}} :\tau ^2 \sim \text{ IG }(q, r)\} = \frac{\Gamma (q + 1/2)\{r + (\theta _r - \theta _o)^2/4\}^{-(q + 1/2)}}{\delta (\theta _r - \theta _o) \, \sqrt{4\pi }}, \end{aligned}$$

so it correctly goes to zero/infinity when the effect sizes $\theta _r$ and $\theta _o$ are equivalent/different. To understand why the test with $\mathcal {H}_{\text {d}} :\tau ^2 \sim \text{ IG }(q,r)$ is consistent, but the original test with $\mathcal {H}_{\text {d}} :\alpha \sim {{\,\textrm{Be}\,}}(1, y)$ is not, one can transform the consistent test on $\tau ^2$ to the corresponding test on $\alpha$. The inverse gamma prior for $\tau ^2$ implies a prior for $\alpha$ with density

23 $$\begin{aligned} f(\alpha \,\vert \,q, r)&= \frac{r^q}{\Gamma (q)} \, \frac{\alpha ^{q - 1}}{(1 - \alpha )^{q + 1}} \, \left( \frac{2}{\sigma ^2_o}\right) ^{q} \, \exp \left\{ -\frac{2 \,r\, \alpha }{\sigma ^2_o(1 - \alpha )}\right\} . \end{aligned}$$

**Fig. 6:**
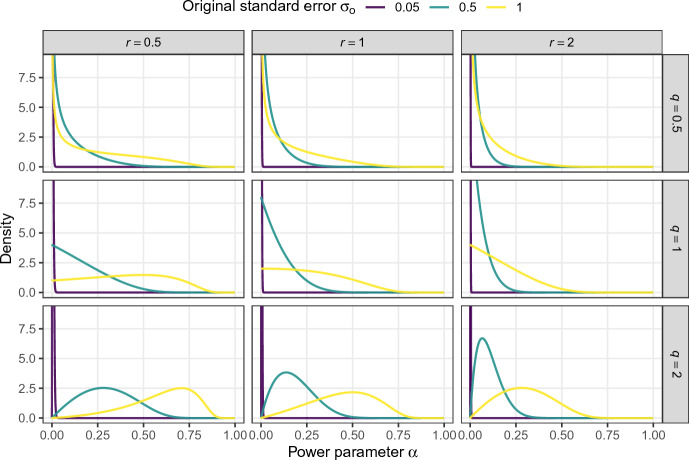
Prior for the power parameter $\alpha$ implied by an inverse gamma prior $\mathcal {H}_{\text {d}} :\tau ^2 \sim \text{ IG }(q, r)$ in a hierarchical model with consistent test for $\mathcal {H}_{\text {c}}:\tau ^{2} = 0$ versus $\mathcal {H}_{\text {d}}:\tau ^{2} > 0$

The Bayes factor contrasting $\mathcal {H}_{\text {c}}:\alpha = 1$ versus $\mathcal {H}_{\text {d}}:\alpha < 1$ with prior (23) assigned to $\alpha$ under $\mathcal {H}_{\text {d}}$ will thus produce a consistent test. The prior is shown in Fig. 6 for different parameters *q* and *r* and original standard errors $\sigma _{o}$. We see that the prior depends on the standard error of the original effect estimate $\sigma _o$, the smaller $\sigma _{o}$ the more the prior is shifted towards zero. For example, the standard error $\sigma _{o} = 0.05$ from the “Labels” experiment leads to priors that are almost indistinguishable from a point mass at $\alpha = 0$. The prior thus “unscales” $\alpha$ from the original standard error $\sigma _o$, thereby leading to a consistent test for study compatibility and resolving the inconsistency property of the beta prior.

## Discussion

We showed how the power prior framework can be used for design and analysis of replication studies. The approach supplies analysts with a suite of methods for assessing effect sizes and study compatibility. Both aspects can be tackled from an estimation or a hypothesis testing perspective, and the choice between the two is primarily philosophical. We believe that both perspectives provide valueable inferences that complement each other. Visualizations of joint and marginal posterior distributions are highly informative in terms of the available uncertainty. However, the power parameter $\alpha$ is an abstract quantity disconnected from actual scientific phenomena. Testing hypotheses of complete discounting versus complete pooling may therefore be more intuitive for researchers. Both approaches also suffer from similar problems: If the original and replication data are in perfect agreement, the posterior distribution of $\alpha$ hardly changes from the prior. For example, for the commonly used uniform prior $\alpha \sim {\textrm{Be}}(x = 1, y = 1)$, we can at best obtain a $\alpha \,\vert \, {\hat{\theta }}_{r} \sim {\textrm{Be}}(x + 1/2 = 3/2, y = 1)$ posterior (Pawel et al. 2023a). This means that for a “compatibility threshold” of, say, 0.8, we can never have a posterior probability higher than ${\textrm{Pr}}(\alpha > 0.8 \,\vert \, {\hat{\theta }}_{r}) = 0.28$, and for a threshold of 0.9 it is even lower ${\textrm{Pr}}(\alpha > 0.9 \,\vert \, {\hat{\theta }}_{r}) = 0.15$. The fact that the Bayes factor for testing $\mathcal {H}_{\text {d}}:\alpha \sim {{\,\textrm{Be}\,}}(1, y)$ against $\mathcal {H}_{\text {c}}:\alpha = 1$ is inconsistent, i.e., bounded from below by a positive constant ${\text {{B}}}(3/2,y)/{\text {{B}}}(1,y)$, simply presents the problem from a different perspective.

We also showed how the power prior approach is connected to hierarchical modeling, and gave conditions under which posterior distributions and hypothesis tests correspond between normal power prior models and normal hierarchical models. This connection provides an intuition for why even with highly precise and compatible original and replication study one can hardly draw conclusive inferences about the power parameter $\alpha$; the power parameter $\alpha$ has a direct correspondence to the relative heterogeneity variance $I^2$, and an indirect correspondence to the heterogeneity variance $\tau ^2$ in a hierarchical model. Making inferences about a heterogeneity variance from two studies alone seems like a virtually impossible task since the “unit of information” is the number of studies and not the number of samples within a study. Moreover, Bayes factor hypothesis tests related to $\alpha$ have the undesirable asymptotic property of inconsistency if a beta prior is assigned to $\alpha$. This is because the prior scales with the variance of the original data, just as a beta prior for $I^2$ would in a hierarchical model. The identified link may also have computational advantages, e.g., it may be possible to estimate power prior models using the hierarchical model estimation procedures, or vice versa, but more research is needed on the connection in more complex situations that depart from normality assumptions.

Which of the two approaches should data analysts use in practice? We believe that the choice should be primarily guided by whether the hierarchical or the power prior model is *scientifically* more suitable for the studies at hand. If data analysts deem it scientifically plausible that the studies’ underlying effect sizes are connected via an overarching distribution then the hierarchical model may be more suitable, particularly because the approach naturally generalizes to more than two studies. On the other hand, if data analysts simply want to downweight the original studies’ contribution depending on the observed conflict, the power prior approach might be more suitable. The identified limitations for inferences related to the power parameter $\alpha$ should, however, be kept in mind when beta priors are assigned to the power parameter $\alpha$.

There are also situations where the hierarchical and power prior frameworks can be combined, for example, when multiple replications of a single original study are conducted (multisite replications). In that case, one may model the replication effect estimates in a hierarchical fashion but link their overall effect size to the original study via a power prior. Multisite replications are thus the opposite of the usual situation in clinical trials where several historical “original” studies but only one current “replication” study is available (Gravestock and Held 2019).

Another commonly used Bayesian approach for incorporating historical data are *robust mixture priors*, i. e.,  priors which are mixtures of the posterior based on the historical data and an uninformative prior distribution (Schmidli et al. 2014). We conjecture that inferences based on robust mixture priors can be reverse-engineered within the framework of power priors through Bayesian model averaging over two hypotheses about the power parameter; however, more research is needed to explore the relationship between the two approaches.

The proposed methods are based on the standard meta-analytic assumption of approximate normality of effect estimates with known variances. This makes our methodology applicable to a wide range of effect sizes that may arise from different data models. However, in some situations this assumption may be inadequate, for example, when studies have small sample sizes. In this case, the methods could be modified to use the exact likelihood of the data (e. g.,  binomial or *t*), as in Bayarri and Mayoral (2002a), who used a *t* likelihood. However, the methodology would need to be adapted for each effect size type. Therefore, future work may examine specific data models in more detail to obtain more precise inferences. In this case, however, using the exact likelihood typically requires numerical methods to evaluate integrals that can be evaluated analytically under normality.

We primarily focused on the evaluation of (objective) Bayesian properties of the proposed methods. Further work is needed to evaluate their frequentist properties, for example, with a carefully planned simulation study (Morris et al. 2019). As in other recent studies (Muradchanian et al. 2021; Freuli et al. 2022), it would be interesting to simulate the realistic scenario of questionable research practices and publication bias affecting the original study to see how the adaptive downweighting of power priors can account for the inflated original results.

## Appendix A: Posterior distribution under the hierarchical model

Under the hierarchical model from (15), the joint posterior conditional on a heterogeneity $\tau ^2$ is given by

24 $$\begin{aligned} f(\theta _{r}, \theta _{o}, \theta _{*} \,\vert \,\hat{\theta }_{o}, \hat{\theta }_{r}, \tau ^{2}) = \frac{\prod _{i \in \{o, r\}} {{\,\textrm{N}\,}}(\hat{\theta }_{i} \,\vert \,\theta _{i}, \sigma ^{2}_{i}) \, {{\,\textrm{N}\,}}(\theta _{i} \,\vert \,\theta _{*}, \tau ^{2}) \, k}{f(\hat{\theta }_{o}, \hat{\theta }_{r} \,\vert \,\tau ^2)} \end{aligned}$$

with normalizing constant

25 $$\begin{aligned} f(\hat{\theta }_{o}, \hat{\theta }_{r} \,\vert \,\tau ^2)&= \int \prod _{i \in \{o, r\}} {{\,\textrm{N}\,}}(\hat{\theta }_{i} \,\vert \,\theta _i, \sigma ^{2}_{i}) \, {{\,\textrm{N}\,}}(\theta _{i} \,\vert \,\theta _{*}, \tau ^{2}) \, k \, \text {d}\theta _o \,\text {d}\theta _r \,\text {d}\theta _* \nonumber \\&= \int \prod _{i \in \{o, r\}} {{\,\textrm{N}\,}}(\hat{\theta }_{i} \,\vert \,\theta _{*}, \sigma ^{2}_{i} + \tau ^2) k \, \text {d}\theta _* \nonumber \\&= k \, {{\,\textrm{N}\,}}(\hat{\theta }_r \,\vert \,\hat{\theta }_o, \sigma ^{2}_{o} + \sigma ^2_r + 2\tau ^2). \end{aligned}$$

To obtain the marginal posterior distribution of the replication effect size $\theta _{r}$ we need to integrate out $\theta _{o}$ and $\theta _{*}$ from (24). This leads to

$$\begin{aligned} f(\theta _{r} \,\vert \,\hat{\theta }_{o}, \hat{\theta }_{r}, \tau ^{2})&= \frac{\int \prod _{i \in \{o, r\}} {{\,\textrm{N}\,}}(\hat{\theta }_{i} \,\vert \,\theta _{i}, \sigma ^{2}_{i}) \, {{\,\textrm{N}\,}}(\theta _{i} \,\vert \,\theta _{*}, \tau ^{2}) \, k \, \text {d}\theta _o \,\text {d}\theta _*}{f(\hat{\theta }_{o}, \hat{\theta }_{r} \,\vert \,\tau ^2)} \\&= \frac{{{\,\textrm{N}\,}}(\hat{\theta }_{r} \,\vert \,\theta _{r}, \sigma ^{2}_{r}) \int {{\,\textrm{N}\,}}(\theta _r \,\vert \,\theta _{*}, \tau ^2) \, {{\,\textrm{N}\,}}(\hat{\theta }_{o} \,\vert \,\theta _{*}, \sigma ^{2}_{o} + \tau ^2) \, \text {d}\theta _*}{{{\,\textrm{N}\,}}(\hat{\theta }_r \,\vert \,\hat{\theta }_o, \sigma ^{2}_{o} + \sigma ^2_r + 2\tau ^2)} \\&= \frac{{{\,\textrm{N}\,}}(\hat{\theta }_{r} \,\vert \,\theta _{r}, \sigma ^{2}_{r}) \, {{\,\textrm{N}\,}}(\theta _r \,\vert \,\hat{\theta }_o, \sigma ^2_o + 2\tau ^2)}{{{\,\textrm{N}\,}}(\hat{\theta }_r \,\vert \,\hat{\theta }_o, \sigma ^{2}_{o} + \sigma ^2_r + 2\tau ^2)} \end{aligned}$$

which can be further simplified to identify the posterior given in (16).

When the heterogeneity $\tau ^2$ is also assigned a prior distribution, the posterior distribution can be factorized in the posterior conditional on $\tau ^2$ from (24) and the marginal posterior of $\tau ^2$

$$\begin{aligned} f(\tau ^2, \theta _{r}, \theta _{o}, \theta _{*} \,\vert \,\hat{\theta }_{o}, \hat{\theta }_{r}) = f(\theta _{r}, \theta _{o}, \theta _{*} \,\vert \,\hat{\theta }_{o}, \hat{\theta }_{r}, \tau ^{2}) \, f(\tau ^2 \,\vert \,\hat{\theta }_{o}, \hat{\theta }_{r}). \end{aligned}$$

Integrating out $\theta _{r}, \theta _{o}$, and $\theta _{*}$ from the joint posterior and using the previous results (25), the marginal posterior of $\tau ^2$ can be derived to be

$$\begin{aligned} f(\tau ^2 \,\vert \,\hat{\theta }_{o}, \hat{\theta }_{r})&= \frac{\int \prod _{i \in \{o, r\}} {{\,\textrm{N}\,}}(\hat{\theta }_{i} \,\vert \,\theta _{i}, \sigma ^{2}_{i}) \, {{\,\textrm{N}\,}}(\theta _{i} \,\vert \,\theta _{*}, \tau ^{2}) \, k \, f(\tau ^2) \, \text {d}\theta _o \,\text {d}\theta _r \,\text {d}\theta _*}{f(\hat{\theta }_o, \hat{\theta }_r)} \\&= \frac{f(\hat{\theta }_r, \hat{\theta }_o \,\vert \,\tau ^2) \, f(\tau ^2)}{\int f(\hat{\theta }_r, \hat{\theta }_o \,\vert \,\tau ^2) \, f(\tau ^2) \, \text {d}\tau ^2} \\&= \frac{{{\,\textrm{N}\,}}(\hat{\theta }_r \,\vert \,\hat{\theta }_o, \sigma ^{2}_{o} + \sigma ^2_r + 2\tau ^2) \, f(\tau ^2)}{\int {{\,\textrm{N}\,}}(\hat{\theta }_r \,\vert \,\hat{\theta }_o, \sigma ^{2}_{o} + \sigma ^2_r + 2\tau ^2) \, f(\tau ^2) \, \text {d}\tau ^2}. \end{aligned}$$

## Appendix B: Conditions for matching posteriors

For the marginal posteriors of $\theta _r$ and $\theta$ to match it must hold for every $\theta$ = $\theta _{r}$ that

26 $$\begin{aligned} f(\theta _r \,\vert \,\hat{\theta }_{o}, \hat{\theta }_{r})&= f(\theta \,\vert \,\hat{\theta }_{o}, \hat{\theta }_{r}) \nonumber \\ \int _{0}^{\infty } f(\theta _{r} \,\vert \,\hat{\theta }_{o}, \hat{\theta }_{r}, \tau ^{2})\, f(\tau ^{2} \,\vert \,\hat{\theta }_{o}, \hat{\theta }_{r})\, \text {d} \tau ^{2}&= \int _{0}^{1} f(\theta \,\vert \,\hat{\theta }_{o}, \hat{\theta }_{r}, \alpha )\, f(\alpha \,\vert \,\hat{\theta }_{o}, \hat{\theta }_{r}) \, \text {d} \alpha . \end{aligned}$$

By applying a change of variables (18) or (19) to the left or right hand side of (26), the marginal posteriors conditional on $\tau ^{2}$ and $\alpha$ match. It is now left to investigate whether there are priors for $\tau ^2$ and $\alpha$ so that also the marginal posteriors of $\tau ^{2}$ and $\alpha$ match. The marginal posterior distribution of $\alpha$ is proportional to

$$\begin{aligned} f(\alpha \,\vert \,\hat{\theta }_{o}, \hat{\theta }_{r}) \propto f_\alpha (\alpha ) \, {{\,\textrm{N}\,}}(\hat{\theta }_{r}\,\vert \,\hat{\theta }_{o}, \sigma ^{2}_{r} + \sigma ^{2}_{o}/\alpha ). \end{aligned}$$

After a change of variables $\tau ^{2} = (1/\alpha - 1)\,(\sigma ^{2}_{o}/2)$ the marginal posterior becomes

$$\begin{aligned} f(\tau ^{2} \,\vert \,\hat{\theta }_{o}, \hat{\theta }_{r}) \propto f_\alpha \left( \frac{\sigma ^{2}_{o}}{2 \tau ^{2} + \sigma ^{2}_{o}}\right) \, \frac{2\sigma ^{2}_{o}}{(2 \tau ^{2} + \sigma ^{2}_{o})^{2}} \, {{\,\textrm{N}\,}}(\hat{\theta }_{r}\,\vert \,\hat{\theta }_{o}, \sigma ^{2}_{r} + \sigma ^{2}_{o} + 2 \tau ^{2}), \end{aligned}$$

Since, as shown in Appendix A, the marginal posterior of $\tau ^2$ under the hierarchical model is proportional to

$$\begin{aligned} f(\tau ^{2} \,\vert \,\hat{\theta }_{o}, \hat{\theta }_{r}) \propto f_{\tau ^2}(\tau ^{2}) \, {{\,\textrm{N}\,}}(\hat{\theta }_{r}\,\vert \,\hat{\theta }_{o}, \sigma ^{2}_{r} + \sigma ^{2}_{o} + 2 \tau ^{2}), \end{aligned}$$

the marginal posteriors of the effect sizes $\theta$ and $\theta _{r}$ match if

$$\begin{aligned} f_{\tau ^2}(\tau ^{2}) = f_\alpha \left( \frac{\sigma ^{2}_{o}}{2 \tau ^{2} + \sigma ^{2}_{o}}\right) \, \frac{2\sigma ^{2}_{o}}{(2 \tau ^{2} + \sigma ^{2}_{o})^{2}} \end{aligned}$$

holds for every $\tau ^{2}\ge 0$.

## Appendix C: The generalized beta and F distributions

A random variable $X \sim \text{ GBe }(a, b, \lambda )$ with density function

27 $$\begin{aligned} f(x\,\vert \,a, b, \lambda ) = \frac{\lambda ^a \, x^{a - 1} \, (1 - x)^{b - 1}}{\text{ B }(a, b) \, \{1 - (1 - \lambda )x\}^{a + b}} \, {\textbf{1}}_{[0, 1]}(x) \end{aligned}$$

follows a generalized beta distribution (in the parametrization of Libby and Novick 1982) with ${\textbf{1}}_{S}(x)$ denoting the indicator function that *x* is in the set *S*. A random variable $X \sim \text{ GF }(a, b, \lambda )$ with density function

28 $$\begin{aligned} f(x\,\vert \,a, b, \lambda ) = \frac{\lambda ^a \, x^{a - 1}}{\text{ B }(a, b) \, (1 + \lambda x)^{a + b}} \, {\textbf{1}}_{[0, \infty )}(x) \end{aligned}$$

follows a generalized F distribution (in the parametrization of Pham-Gia and Duong 1989).

## Software and data

The CC-By Attribution 4.0 International licensed data were downloaded from https://osf.io/42ef9/. All analyses were conducted in the R programming language version 4.3.0 (R Core Team , 2020). The code and data to reproduce this manuscript is available at https://github.com/SamCH93/ppReplication. A snapshot of the GitHub repository at the time of writing this article is archived at https://doi.org/10.5281/zenodo.6940237. We also provide an R package for estimation and testing under the power prior framework https://CRAN.R-project.org/package=ppRep. The package can be installed by running install.packages("ppRep") from an R console.
